# Linking shox/shox2 deficiency with fgfr3 gain-of-function and natriuretic peptides

**DOI:** 10.3389/fendo.2026.1803846

**Published:** 2026-04-17

**Authors:** Sandra Hoffmann, Sabrina Diebold, Ralph Roeth, Annette Löwen, Stefanie Mellein, Steffen Just, Gudrun A. Rappold

**Affiliations:** 1 Institute of Human Genetics, University Hospital Heidelberg, Heidelberg, Germany; 2 DZHK (German Centre for Cardiovascular Research), Partner Site Heidelberg/Mannheim, Heidelberg, Germany; 3 Molecular Cardiology, Clinic for Internal Medicine II, University Hospital Ulm, Ulm, Germany; 4 Expression & Spatial Profiling Core Facility, Institute of Human Genetics, University Hospital Heidelberg, Heidelberg, Germany

**Keywords:** ANP, BNP, CNP, FGFR3, natriuretic peptides, short stature, SHOX, SHOX2

## Abstract

Short stature homeobox (*SHOX)* and its close paralogue SHOX2 are important transcriptional regulators of developmental processes. To understand how they function both individually and as an ensemble, we examined their mutual interdependence using zebrafish as a model. As both genes maintain important roles in various tissues, we assessed their expression patterns in the whole embryo as well as separately in fins, heart and brain. Fibroblast growth factor receptor 3 (*FGFR3)* is a direct target of SHOX, and *FGFR3* mutations cause achondroplasia, a condition treatable with C-type natriuretic peptide (CNP) analogues. Building on this knowledge, we investigated how altered *shox* and *shox2* expression relates to *fgfr3* expression and the natriuretic peptide family (*nppa*, *nppb*, *nppc*) in zebrafish. Here, we demonstrate that *shox* and *shox2* mutually regulate each other’s expression in a tissue-specific manner. In brain and whole embryos their effects are additive and synergistic, while in fins and heart their combined actions become partially antagonistic. Distinct tissue-specific effects of *shox* and *shox2* were also noted on natriuretic peptide gene activity. In the fin, *shox* knockdown reduces *nppc* transcript levels, whereas in heart tissue, deficiency of *shox* and *shox2*, particularly in the double knockdown, results in an increased expression of *nppb*, a known marker for cardiac stress. We also show that shox2 deficiency increases *fgfr3* levels, which are reduced by CNP treatment. These results in zebrafish may have implications for other vertebrates, including humans with SHOX deficiency, who should be monitored for cardiac disease in later life, and those who have an inadequate response to growth hormone may profit from CNP therapy.

## 1 Introduction

SHOX and SHOX2 belong to a distinct subfamily of homeobox transcription factors. Both proteins are known for their different and shared developmental functions and spatial sub- functionalization, and clinical phenotypes. SHOX was initially identified as the central component for the short stature phenotype in Turner syndrome (45,X) (1, 2). Isolated (idiopathic) and syndromic (Leri-Weill dyschondrosteosis; Langer syndrome) short stature phenotypes are also caused by *SHOX* variants, deletions of the gene or its enhancer regions (3, 4). *SHOX* duplications usually lead to tall stature or have been associated with a Club foot-like deformity, supporting the notion that particularly bone/cartilage and the skeleton are affected by an abnormal *SHOX* expression (5). Taken all these clinical phenotypes into account, SHOX deficiency with different expression patterns and severities likely represents the most common cause of monogenic short stature, affecting the length of the distal limbs, as well as other skeleton-muscular features. Yet, given that this gene is expressed in diverse embryonic, fetal and adult tissues as well as organs including heart and brain - and given its broader function as a transcription factor - other phenotypic consequences besides skeletal deficits cannot be ruled out (6, 7).

SHOX2 has been identified due to its close homology to SHOX and mutations in this gene cause cardiac phenotypes with oedema and bradycardia in mouse and zebrafish, and atrial fibrillation and sinus node dysfunction in humans (8–11). Besides the cardiac phenotype, the knockout of *Shox2* in mice leads to shortened proximal limbs, affecting primarily femur and tibia skeletal elements (12, 13).

Given that both genes, *SHOX* and *SHOX2*, share and encode an identical protein structure, the homeodomain, that binds DNA to regulate expression of target genes, partly similar regulatory effects are to be expected. However, it is not known whether the SHOX protein has a regulatory effect on *SHOX2* expression and *vice versa*. We can test this assumption in zebrafish, a broadly used vertebrate animal model. Unlike the mouse, zebrafish offers the appropriate biological context, where both *shox* and *shox2* are present and expressed. This also provides the opportunity to assess if all the members of the natriuretic gene family, *nppa* (ANP), *nppb* (BNP) and *nppc* (CNP) are regulated by Shox and Shox2, as previously shown for the direct *SHOX* target gene *NPPB* (BNP) (14, 15). ANP and BNP are well-known cardiac natriuretic peptide hormones; BNP (or its N-terminal fragment NT-proBNP) in the plasma is used as a diagnostic and prognostic biomarker for heart failure (16). C-type natriuretic peptide CNP (*NPPC*) on the other hand is a potent stimulator of endochondral ossification leading to improved bone growth in children with achondroplasia by antagonizing fibroblast-growth-factor-receptor-3 (FGFR3) downstream signaling (17). As SHOX directly binds to and regulates *FGFR3* as a target (18), there is an interest to clarify the interrelation between SHOX and SHOX2, as well as between *SHOX*/*SHOX2*, *FGFR3* and the natriuretic gene family.

Here, we performed expression profiling in zebrafish to investigate the regulatory potential of *shox* on *shox2*, and vice versa across different tissues. Using this animal model, we can demonstrate how changes in the expression of one gene affect the other, providing new insights into their mutual regulation and their roles in developmental processes. Moreover, we show that shox2-deficient embryos exhibit increased *fgfr3* levels, which are restored by CNP treatment.

## 2 Methods

### 2.1 Zebrafish embryos and microinjections

Zebrafish (*Danio rerio*) were maintained as previously described (19). For morpholino injection experiments followed by tissue-specific analyses, the Tg(myl7:GFP) strain was used (20). All other morpholino injections were performed in the TüAB wildtype strain. Morpholino-modified antisense oligonucleotides (MO; Gene Tools) were directed against the exon 2 intron 2 junction in *shox*, causing reduction of pectoral fins as described previously (21). MO were directed against the splice donor site of *shox2* in intron 3, causing the same bradycardia phenotype as the *shox2* MO directed against the translational start site. Both MOs have been described previously (9, 22). The *fgfr3* MO was directed against the splice donor site of *fgfr3* in intron 1. For the *fgfr3* splice-blocking MO, RT-PCR analyses spanning the targeted exon-intron boundaries were performed to confirm aberrant splicing. Gel electrophoresis verified the expected exon skipping event (**Supplementary Figure 1A**). Mis-splicing induced by *shox* or *shox2* morpholino knockdown was previously validated (9, 21).

*Shox* (200 µM) and *shox2* (300 µM) antisense oligonucleotides together with a standard control oligonucleotide (MO control), diluted in 0.2 M KCl, were microinjected into one-cell-stage zebrafish embryos (23).

Briefly, morpholinos (shox (200 µM) and shox2 (300 µM)) were diluted in Danieau buffer containing 0.05% phenol red and injected into the yolk of one-cell stage embryos using calibrated injection needles delivering ~1–2 nL per embryo. Injection volume was regularly validated using a micrometer scale. Morpholinos were heat-denatured at 65 °C for 10 minutes prior to use to ensure proper solubilization. Aliquots were stored at -20 °C to prevent repeated freeze-thaw cycles. Only embryos that exhibited the respective phenotype after knockdown were considered for further analysis.

For combined injections, morpholinos were premixed prior to loading to ensure uniform delivery. Different *fgfr3* MO concentrations between 300 µM and 5 µM were tested to find a concentration that produces a detectable splice effect but does not induce a phenotype (**Supplementary Figure 1**). The desired effect was finally achieved at a concentration between 100 - 5 µM. Sequence information for all Morpholino-modified antisense oligonucleotides is summarized in **Supplementary Table 1**.

Toxicity controls were performed for all MO applications. Embryos were systematically evaluated for developmental delay, necrosis, cardiac edema, and axis malformations. For combined MO injections, total MO concentrations were titrated to remain within established non-toxic ranges based on previously published and internally validated conditions. No increase in general toxicity or non-specific malformations was observed compared to single MO injections at equivalent cumulative concentrations.

Experiments were performed exclusively on zebrafish embryos and larvae up to 5 days post fertilization. At this developmental stage, zebrafish are not considered protected animals under the relevant animal welfare legislation (e.g., EU Directive 2010/63/EU and corresponding national regulations), and therefore the procedures do not constitute regulated animal experiments requiring formal ethical approval. Consequently, no institutional ethics statement was required for the work presented here.

### 2.2 Treatment with CNP

For CNP experiments, embryos were treated at 1 hour post-fertilization (hpf) with different concentrations of CNP (Natriuretic Peptide, C-Type, Human and Porcine, Sigma-Aldrich, N8768 and Bio-Techne, 3520/500U), ranging from 25 nM to 10 µM (25, 50, 75, 100, 150, 200, 300, 400, 500, 600, 700, 800, 900 nM, and 1, 1.2, 1.5, 2, 2.5, 3, 3.5, 4, 4.5, 5, 5.5, 6, and 10 µM). Peptide handling strictly followed the manufacturers’ recommendations, including aliquoting (aliquots of 100 µM stock solution solved in H_2_O) and storage at −80 °C. All working solutions were freshly prepared from aliquots and were not subjected to repeated freeze–thaw cycles. The treatment was maintained until 72 hpf, with the CNP solution freshly prepared and replaced daily.

### 2.3 nCounter expression analysis

Zebrafish tissues were isolated at 55 hpf and total RNA was extracted with the Direct-zol RNA Microprep Kit (Zymo Research) according to the manufacturer’s instructions. For each experiment, whole zebrafish embryos (10–15), hearts (30–70), heads (~20), or pectoral fins (30–40) were pooled per condition to obtain 50 ng of input material. Three to six independent experiments (n) were performed. mRNA expression levels were measured at the Expression & Spatial Profiling Core Facility Heidelberg using the nCounter SPRINT Profiler. This RNA quantification technology utilizes a direct digital detection of mRNA molecules and allows multiplexed target measurement in a single reaction with high sensitivity and specificity even with low amounts of input material (50 ng). A detailed probe design is given in **Supplementary Table 1**. The workflow is described at http://www.nanostring.com/elements/workflow. The nCounter customized codeset included 6 negative controls for background substraction, 6 positive controls for lane to lane and cartridge to cartridge normalization, 6 reference genes (*actb1*, *b2m*, *eef1a1l1*, *hsp90ab1*, *rpl13a*, *rps18*) for mRNA normalization and 6 genes of interest (*shox*, *shox2*, *fgfr3*, *nppa*, *nppb*, *nppc*). Since zebrafish can have several copies of the same gene, we analyzed the predominantly expressed copies that represent orthologues to humans. Normalization of data was performed using the nSolver Analysis Software 4.0 (NanoString Technologies). Most stable expressed reference genes were chosen for normalization based on the NormFinder algorithm (24). Normalized nCounter datasets are provided in **Supplementary Table 2**.

### 2.4 Statistical analyses

Statistical analysis was carried out using GraphPad Prism 10 (GraphPad Software, La Jolla, CA, United States). For multiple t-tests, the two-stage step-up method of Benjamini, Krieger, and Yekutieli with a desired false discovery rate (FDR) of 5% was used to correct the values of p. All conditions statistically different from the control were indicated by *P < 0.05; **P < 0.01; ***P < 0.001 and ****P < 0.0001. Multiple comparisons were performed using a two-way ANOVA followed by Tukey’s multiple comparison test with adjusted p-values.

## 3 Results

### 3.1 Shox knockdown affects shox2 expression and vice versa

In the developing zebrafish embryo, *shox* is known to be expressed in different tissues including pectoral fins, heart, brain and pharyngeal arches (25, 26). We have now analyzed expression of its paralog *shox2*, and quantified *shox* and *shox2* levels in four selected tissues - fin, heart, head (including brain, pharyngeal arches, eye, and olfactory epithelium) as well as in whole embryo. For simplicity, we will use the term *brain* instead of *head* during the entire manuscript in the text from now on.

**Figure 1:**
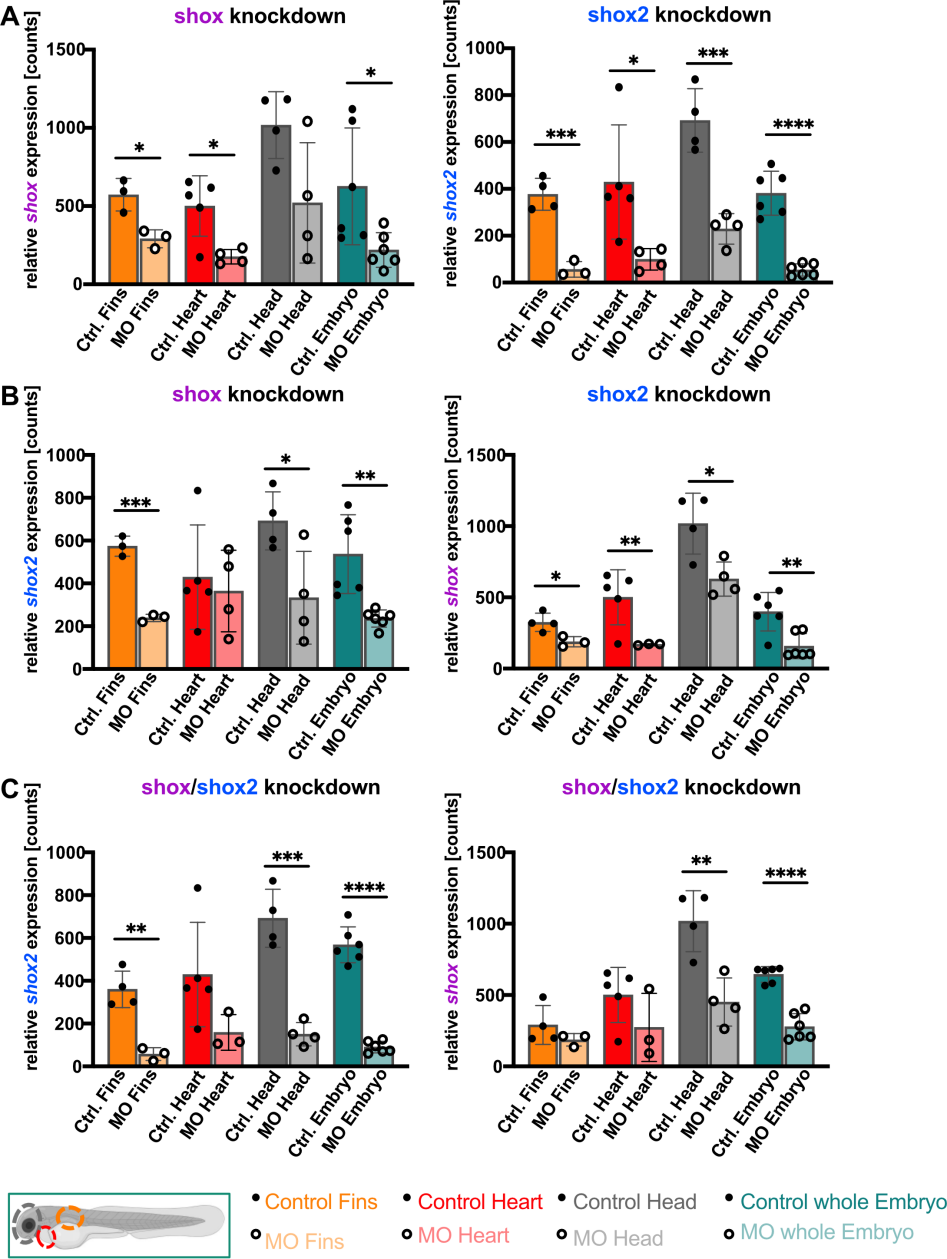
*Shox* knockdown affects *shox2* activity and vice versa: Tissue-specific nCounter analysis of *shox* and *shox2* expression in zebrafish embryos 55 hpf upon *shox* and *shox2* single knockdown **(A, B)** and *shox*/*shox2* double knockdown **(C)** in fin (n=3), heart (n=4-5), head (n=4) and whole embryo (n=6). *P < 0.05, **P < 0.01, ***P < 0.001, ****P < 0.0001. MO = morpholino, hpf = hours post fertilization, n = number of independent experiments.

We then compared expression levels in these tissues between normal and knockdown conditions. Knockdown for *shox* and *shox2* was robust and efficient in all tested tissues, with a higher knockdown efficiency for *shox2* compared to *shox* (**Figure 1A**). Our results demonstrate that knockdown of *shox* significantly reduces *shox2* expression levels in fins, brain and whole embryo, but not in the heart. In heart tissue, the *shox* knockdown does not show a significant effect on *shox2* levels. *Shox2* knockdown, however, leads to significantly reduced *shox* levels in all analyzed tissues (fin, heart, brain and whole embryo) including the heart (**Figure 1B**). This data demonstrates that both genes influence each other in a dependable way (with the possible exception of heart tissue).

Then we asked whether the double knockdown of *shox*/*shox2* mitigates or strengthens these effects. In the double knockdown of *shox*/*shox2*, the expression of both *shox* and *shox2* in brain and whole embryo is further reduced and thus the reduction reinforced. In the fins, *shox* and *shox2* levels were not further reduced by the double knockdown, suggesting that *shox* and *shox2* might have partly antagonizing effects in the fins (**Figure 1C**). In the heart, the double knockdown does not lead to a significant change in *shox2* levels, suggesting that *shox* does not significantly interfere with *shox2* expression levels in the heart. This is also indicated by the fact that a cardiac phenotype has not been observed in zebrafish after *shox* knockdown (7). In the *shox2* morphants, however, *shox* transcript level in heart tissue is significantly reduced (**Figure 1B**), while the *shox*/*shox2* double knockdown seems to equilibrate this effect (**Figure 1C**).

These data illustrate the interrelation and mutual dependence of Shox and Shox2, suggesting that in brain and whole embryos their effects are additive and synergistic, while in fins and heart their combined actions become partially antagonistic and consequently weakened.

### 3.2 Shox and shox2 knockdown and their effects on the transcript levels of natriuretic peptide genes

**Figure 2:**
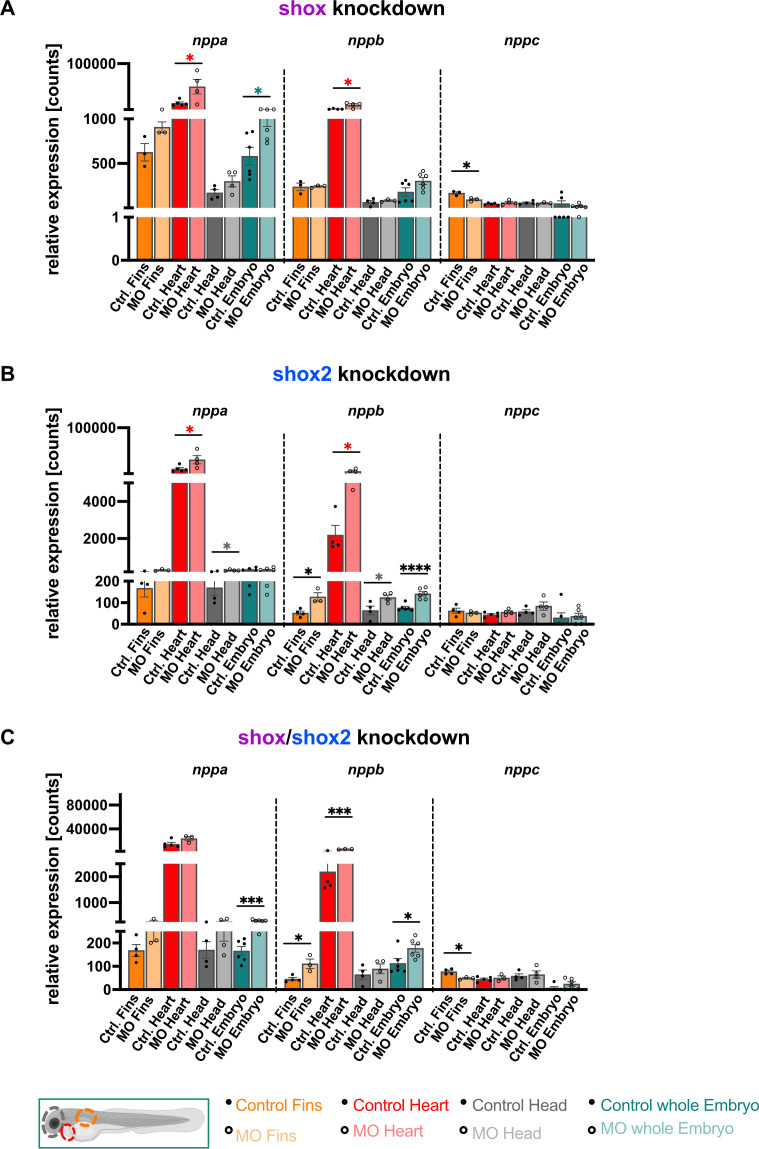
*Shox* and *shox2* knockdown affect expression levels of the natriuretic peptide genes *nppa*, *nppb* and *nppc* in a tissue-specific manner. Relative expression levels upon *shox* **(A)**, *shox2* **(B)** and *shox*/*shox2* **(C)** knockdown in fin (n=3), heart (n=4-5), head (n=4) and whole embryo (n=6). Statistical significance was determined by multiple unpaired *t*-tests adjusted by the two-stage step-up method of Benjamini, Krieger, and Yekutieli (adjusted values of *P* are indicated as black asterisks, nominal significant values of *P* are indicated as colored asterisks). *P < 0.05, **P < 0.01, ***P < 0.001, ****P < 0.0001). MO = morpholino, hpf = hours post fertilization, n = number of independent experiments.

To examine a putative regulatory effect of *shox* and *shox2* on the natriuretic peptide gene family consisting of *nppa* (ANP), *nppb* (BNP) and *nppc* (CNP), tissue-specific expression levels were analyzed under normal and knockdown conditions. In *shox* morphants, *nppa* levels were significantly elevated in whole embryos; in the heart, *nppa a*s well as *nppb* levels were significantly increased, while in the fin, *nppc* expression levels were reduced (**Figure 2A**). In *shox2* morphants, *nppa* and *nppb* levels were significantly elevated in the heart, with *nppa* also showing increased expression in the brain. *Nppb* levels were also elevated in the fin, brain, and whole embryo, whereas no changes were observed for *nppc* expression (**Figure 2B**).

In the *shox*/*shox2* morphants, *nppa* levels were significantly increased in whole embryo, suggesting that *shox* and *shox2* have additive effects and re-enforce each other expression in the zebrafish embryo. In heart tissue, a similar effect was observed for *nppb*: *shox*/*shox2* knockdown caused significantly higher *nppb* levels, while this was not the case for *nppa* levels (**Figure 2C**).

In the fin, the expression of *nppb* and *nppc* but not of *nppa* is significantly affected by the *shox*/*shox2* knockdown. While the increased *nppb* expression may reflect the effect of the *shox2 knockdown*, the reduced *nppc* expression observed in the double knockdown is likely primarily caused by the *shox* knockdown (**Figure 2C**).

These data show distinct tissue-specific effects of *shox* and *shox2* on natriuretic peptide gene levels following knockdown. In the fin, *shox* knockdown reduces *nppc* transcript levels, whereas in heart tissue, deficiency of *shox* and *shox2*, particularly in the double knockdown, results in an increased expression of *nppb*, a known marker for cardiac stress and heart failure.

### 3.3 Shox/shox2 knockdown and consequences on fgfr3 transcript levels

Gain-of-function mutations in *FGFR3* underlie achondroplasia (27, 28). In the growth plate, FGFR3 signaling is counteracted by C-type natriuretic peptide CNP via natriuretic peptide receptor-B (NPR-B, also termed NPR-2). Since *FGFR3* is a direct downstream target of SHOX (18), we investigated how knockdown of *shox* or *shox2* - individually and in combination - modulates *fgfr3* expression in the analyzed tissues.

**Figure 3:**
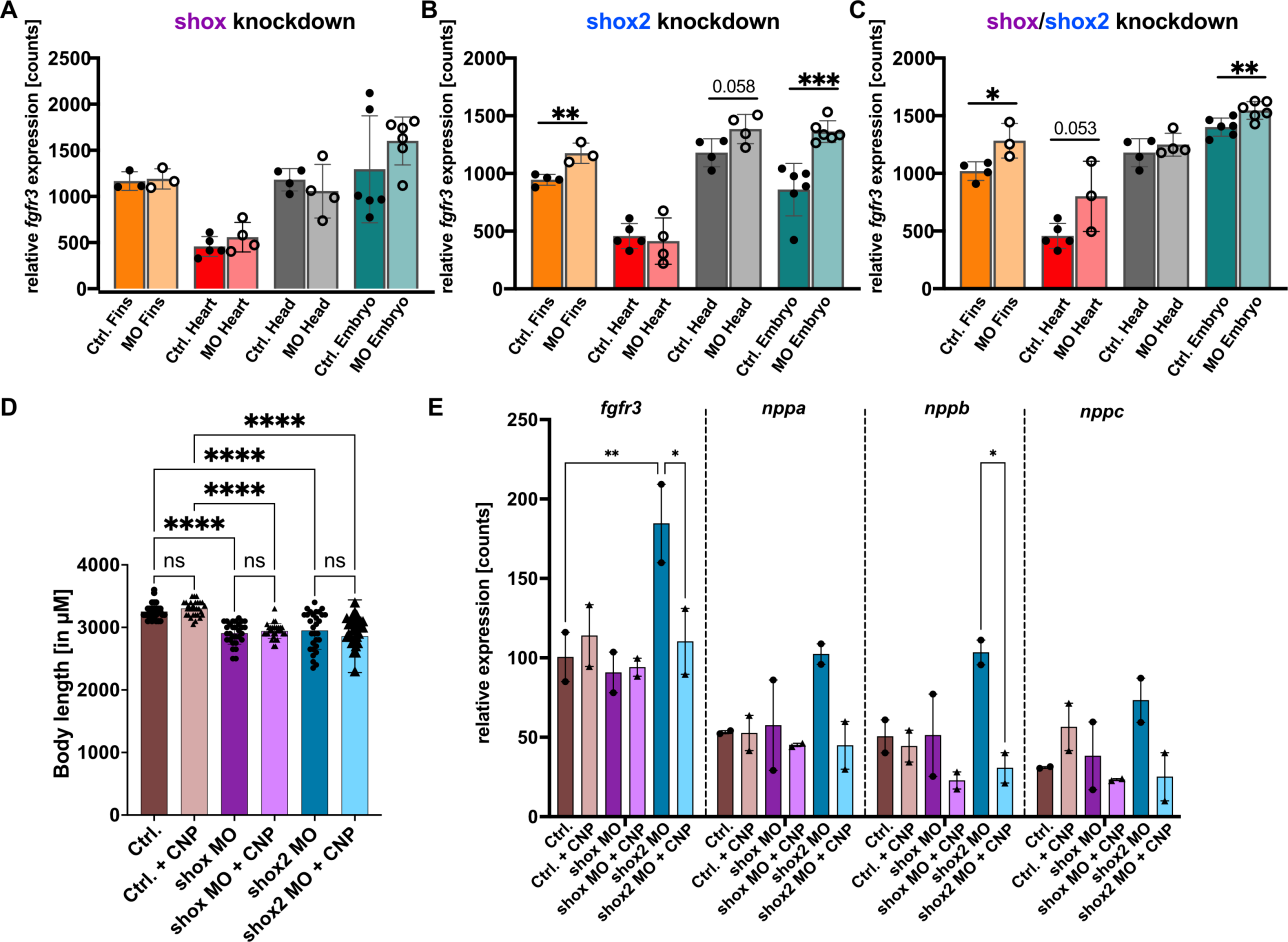
Tissue-specific nCounter expression analysis of *fgfr3* in zebrafish embryos 55 hpf upon *shox* **(A)**, *shox2* **(B)** and *shox*/*shox2* **(C)** knockdown in fin (n=3), heart (n=4-5), head (n=4) and whole embryo (n=6). MO = morpholino. **(D)** Body length of zebrafish embryos 72 hpf after *shox* and *shox2* knockdown and CNP treatment (10 µM), assessed in 10–15 whole embryos per condition (n=3). **(E)** nCounter expression analysis of *fgfr3, nppa, nppb, nppc* in zebrafish embryos 72 hpf after *shox* and *shox2* knockdown and CNP treatment (10µM), assessed in 10–15 whole embryos per condition (n=2). *P < 0.05, **P < 0.01, ***P < 0.001, ****P < 0.0001. MO = morpholino, hpf = hours post fertilization, n = number of independent experiments.

*Shox* knockdown led to minimal changes in *fgfr3* expression across the tissues examined, possibly also caused by the less efficient knockdown of *shox* (**Figure 3A**). In contrast, *shox2* knockdown elicited a robust and significant upregulation of *fgfr3* in fins and whole embryos (**Figure 3B**). A similar effect was observed in the combined *shox*/*shox2* knockdown, leading to significantly elevated *fgfr3* levels in fin and whole embryo tissues, with a (moderate) increase also detected in heart (**Figure 3C**).

To investigate if *shox2* through the regulation of *shox* (or *shox* through the regulation of *shox2*) exerts an effect on *fgfr3* expression, we assessed whether the limb phenotype in *shox* and *shox2* morphants is altered by *fgfr3* expression. For this purpose, we considered experiments, in which an additional *fgfr3* knockdown in *shox* or *shox2* morphants was induced. We titrated the splice-blocking *fgfr3* morpholino (MO) to determine a concentration that preserved efficient splice interference, while avoiding induction of a morphological phenotype (300, 125, 100, 50, 25, 10, 5 µM). Higher concentrations of 300 µM proved toxic and 125 µM elicited a phenotype marked by reduced birefringence, consistent with muscle damage, smaller fins, reduced lower jaw, atrophied tail tip and bradycardia (**Supplementary Figures 1A, B**). In contrast, embryos single-injected with 5-100 µM *fgfr3* exhibited normal, wildtype morphology.

Co-injection of *shox* (200 µM) and *fgfr3* (100 and 50 µM) MOs resulted in a markedly enhanced phenotype (atrophied tail tip, extremely reduced fins size, bradycardia, cerebral oedema) compared with the *shox* single-knockdown, indicating an additive effect between *shox* and *fgfr3* (**Supplementary Figure 1C**). Even at a reduced *fgfr3* MO dosis (25 and 10 µM), the combined knockdown produced consistently stronger phenotypes. Similar additive effects were also observed in the double knockdown embryos of *shox2* (300 µM) and *fgfr3* (100 µM). To further characterize the impact of *fgfr3* depletion in *shox2* morphants, we titrated the *fgfr3* MO to even lower concentrations (10 and 5 µM), yielding a subset of embryos displaying wildtype morphology (**Supplementary Figures 2A, B**). To discern whether this phenotype reflected a genuine rescue effect or reduced penetrance of the *shox2* knockdown, we conducted additional molecular analyses to confirm *shox2* knockdown efficiency. nCounter expression analyses revealed that embryos with wildtype morphology, whether injected with the *shox2* MO alone or together with the *fgfr3* MO, maintained *shox2* transcript levels comparable to controls, consistent with only partial knockdown or a reduced morpholino efficacy. By contrast, *shox2* morphants manifesting the characteristic phenotype – reduced fin size and bradycardia - showed a clear reduction in *shox2* expression (**Supplementary Figure 2C**).

### 3.4 CNP treatment of shox and shox2 morphants and consequences on fgfr3 transcript levels

To further interrogate FGFR3 signaling, we next attempted pharmacological modulation using CNP treatment. CNP acts as an antagonist of FGFR3, and as shown in **Figure 2A**, shox deficiency in zebrafish fins markedly reduces *nppc* levels. Using a CNP analogue (Sigma-Aldrich) previously used in zebrafish studies (29), we tested multiple concentrations of the compound across a broad range (25, 50, 75, 100, 150, 200, 300, 400, 500, 600, 700, 800, 900 nM up to 1 - 1.2 - 1.5 - 2 - 2.5 - 3 - 3.5 - 4 - 4.5 - 5 - 5.5 - 6 - 6.5 - 10 µM). However, even at the highest concentration administered (10 µM), treated embryos were indistinguishable from untreated controls, exhibiting no morphological alterations or detectable signs of toxicity (**Figure 3D**). Nonetheless, we treated *shox* and *shox2* morphants with a high concentration of 10 µM CNP to assess potential morphological or molecular effects. No significant morphological differences were observed between treated and untreated morphants (**Figure 3D**), suggesting that the administered CNP was not fully functional. However, nCounter molecular expression analysis revealed that the elevated *fgfr3* levels observed in *shox2* morphants (as previously shown in independent experiments, **Figure 3B**) were normalized upon CNP treatment (**Figure 3E**). These findings suggest that *shox*/*shox2*-deficient embryos exhibit increased *fgfr3* levels, which can be restored by the natriuretic peptide CNP.

## 4 Discussion

We have analyzed the functional interplay between *shox*, *shox2*, *fgfr3* and the three natriuretic peptide genes *nppa*, *nppb*, *nppc* in zebrafish. Zebrafish was selected as a vertebrate model because all genes of interest are present and expressed, and their expression levels can be readily measured. We examined zebrafish at 55 and 72 hours post-fertilization (hpf), which corresponds to very early postnatal or neonatal stages in mammals (30). By this stage, the biological basis of growth deficits is already established, even if clinical manifestation or diagnosis occurs later in childhood.

*SHOX* and *SHOX2* encode transcription factors with various functions during development whose expression are tightly regulated by different promotors and multiple cis-regulatory enhancers (4, 31). This enables expression of the *SHOX* and *SHOX2* genes in multiple tissues and different developmental structures. Temporal specificity of enhancers ensures precise orchestration of gene expression across developmental stages (32). Moreover, the availability and interactions of cofactors, including coactivators and corepressors, differ among tissues, leading to distinct cell-specific regulatory effects. CRISPR-mediated mutant shox2 fish, for example, have been shown to be required for vestibular statoacoustic neuron development (33), while shox^-/-^ zebrafish exhibit impaired bone growth (34). In our study, this tissue-specific activity was taken into account by analyzing not only whole zebrafish embryos but also fin, heart, and brain (head) tissues separately, all of which exhibited characteristic and distinct expression patterns.

Redundancy between SHOX and SHOX2 has been observed in different biological systems before. For example, immunohistochemistry of human fetal growth plates from different time points demonstrated that *SHOX2* is co-expressed with *SHOX* (15). In luciferase reporter assays, it was demonstrated that SHOX2, like SHOX, regulates *NPPB* (14, 15). Consistently, significant deregulation of *Nppb* was observed in *Shox2*-deficient mouse heart tissue and in embryonic stem cell–derived cardiomyocytes (35), mirroring the effects observed in zebrafish in this study. In the heart, functional redundancy was demonstrated between human *SHOX* and mouse *Shox2* genes in the regulation of sinoatrial node formation and pacemaking function (36).

In this study, we identified a reciprocal dependency between *shox* and *shox2*, with each influencing the expression of the other. Our data also reveal that *shox* deficiency, as well as the combined shox/shox2 knockdown in zebrafish fins, leads to a significant reduction in *nppc* levels. These findings suggest that individuals with SHOX (and SHOX2) deficiency may benefit from an increased CNP activity. In addition, we demonstrate that *shox2* knockdown in zebrafish results in elevated *fgfr3* levels. Yasoda et al. were the first to show that targeted overexpression of CNP in chondrocytes counteracts the dwarfism phenotype in a mouse model of achondroplasia by inhibiting the MAPK signaling pathway downstream of activated Fgfr3. These results supported the idea that activating the CNP-Guanylylcyclase-B system could be a novel strategy for achondroplasia (37).

Our initial attempt to rescue the limb phenotype in *shox* and *shox2* morphants through double knockdown experiments including *fgfr3* was unsuccessful, likely due to several reasons: double morpholino knockdowns are inherently challenging because of variability in morpholino efficiency, differences in target gene expression levels, and potential off-target effects. Furthermore, compensatory mechanisms or genetic redundancy in zebrafish may mask the expected phenotypic rescue (38). Even in our well-established morpholino experiments targeting *shox* and *shox2* (7, 10, 11, 22, 39) we observed variable penetrance, as indicated by expression analysis following phenotypic assessment. For example, some *shox2* morphants displaying a wildtype appearance did not show reduced *shox2* expression levels, whereas those with a pronounced phenotype showed clear downregulation. This variability is also the reason why morphants were selected based on phenotype prior to tissue-specific isolation and gene expression profiling.

Notably, we demonstrate that elevated *fgfr3* levels in *shox2* morphants can be restored to baseline by CNP treatment. For our experiments, we employed two commercially available CNP analogues, obtained from Sigma-Aldrich and Bio-Techne, because the long-acting, chemically modified CNP analogue by BioMarin Pharmaceutical (Vosoritide, marketed as VOXZOGO^®^) was unfortunately not accessible for our experiments. Consequently, we anticipate that an extended-half-life CNP analogue would have elicited even more robust and sustained biological effects.

**Figure 4:**
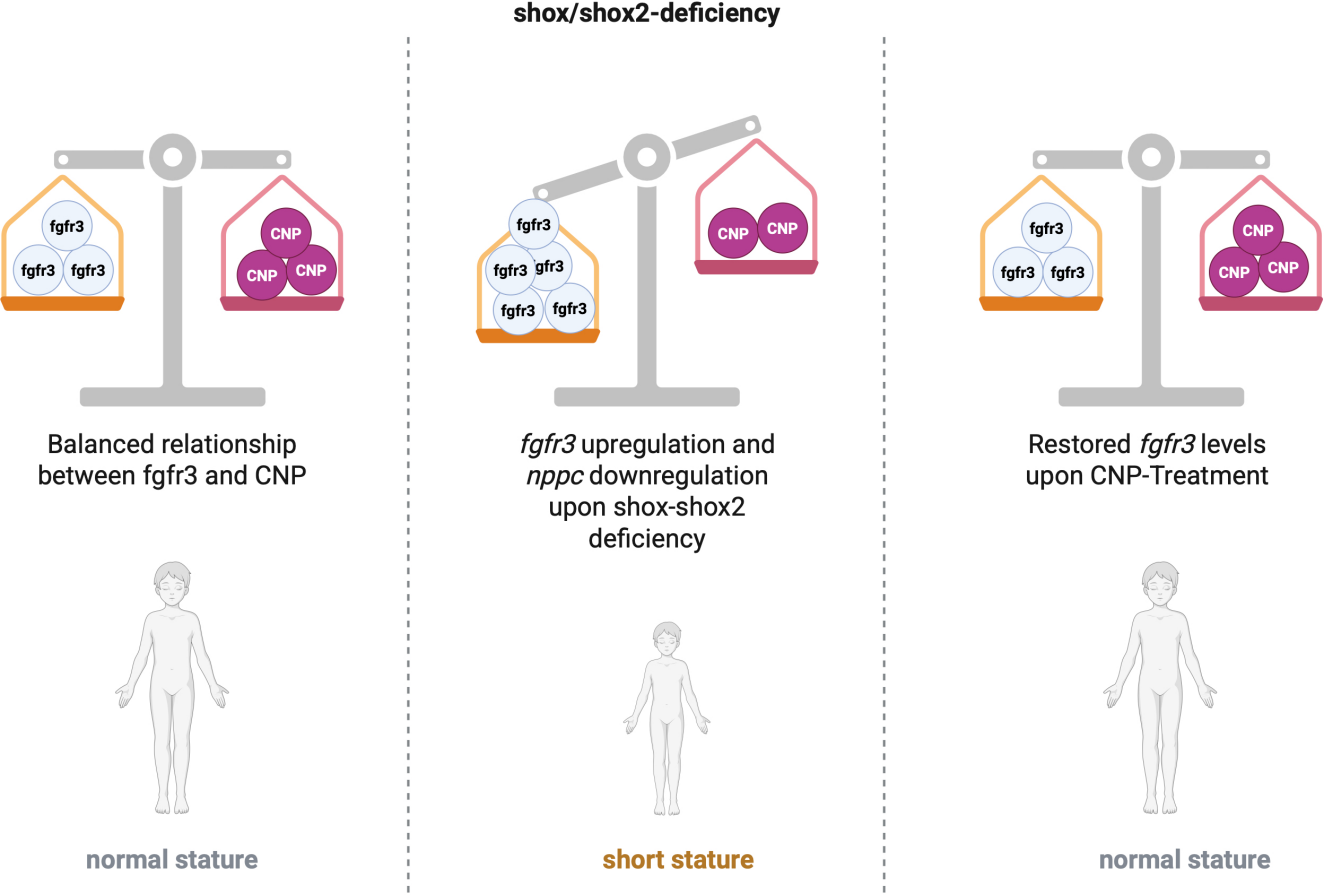
Schematic hypothesis illustrating the relationship between *fgfr3* expression and CNP in shox/shox2 deficiency, based on molecular analyses in zebrafish. Created with BioRender.

Achondroplasia is driven by an excessive FGFR3 activity; the underlying problem, however, is not a deficiency of CNP, but rather that physiological CNP levels are insufficient to counterbalance the heightened FGFR3 signaling (**Figure 4**). The concept that CNP could serve as a therapeutic agent initially arose from observations that loss-of-function variants in its receptor, NPR-B, cause severe short stature, whereas CNP overexpression leads to skeletal overgrowth (40–42). These findings indirectly suggested that augmenting CNP signaling – via CNP binding to the NPR-B receptor and subsequent modulation of the MAPK pathway – could suppress FGFR3 activity. Consequently, pharmacologic CNP analogues with extended half-lives (e.g. Vosoritide) have been developed and are now successfully used in the treatment of achondroplasia (ACH) and hypochodroplasia (HCH) (43–46). In addition, there is an ongoing clinical trial (NCT06668805), testing Vosoritide in children with Turner and Noonan syndrome and children with SHOX deficiency who have an inadequate response to growth hormone, but there is no direct published clinical evidence yet (47).

If we extrapolate the findings of our zebrafish study to humans, several implications may be considered for individuals with SHOX deficiency. Given that SHOX modulates *SHOX2* expression and that mutations in *SHOX2* have been implicated in cardiac pacemaker function, individuals with SHOX deficiency may also have a higher risk of developing arrhythmias. Given the increased BNP levels in the heart following *shox* and *shox2* knockdown, it is also reasonable to assume that individuals with SHOX deficiency are at an increased risk of general heart problems, particularly in old age. An increased cardiovascular mortality has been noted in children with growth hormone deficiency and intrauterine growth retardation before (48, 49). Furthermore, the observation that shox knockdown results in significantly reduced *nppc* levels, while *shox2* knockdown leads to significantly elevated *fgfr3* levels, provides support for the benefit of CNP therapy also in individuals with SHOX/SHOX2 deficiency. Further data and additional studies in individuals with SHOX deficiency will be needed to validate these assumptions.

## Data Availability

The original contributions presented in the study are included in the article/**Supplementary Material**. Further inquiries can be directed to the corresponding authors.
