# Supplementary material for: Linking shox/shox2 deficiency with fgfr3 gain-of-function and natriuretic peptides
Source: Front Endocrinol (Lausanne). 2026 Apr 17;17:1803846. doi: 10.3389/fendo.2026.1803846 (PMC13132733; doi:10.3389/fendo.2026.1803846)
Supplement: Supplementary file 1 [file DataSheet1.docx]

**Supplementary Figures**

**Sandra Hoffmann^1,2^, Sabrina Diebold^3^, Ralph Roeth^1,4^, Annette Löwen^1^, Stefanie Mellein^1,4^, Steffen Just^3^, Gudrun A. Rappold^1,2^**

^1^Institute of Human Genetics, University Hospital Heidelberg, Heidelberg, Germany

^2^DZHK (German Centre for Cardiovascular Research), Partner Site Heidelberg/Mannheim, Heidelberg, Germany

^3^Molecular Cardiology, Clinic for Internal Medicine II, University Hospital Ulm, Ulm, Germany

^4^ Expression & Spatial Profiling Core Facility, Institute of Human Genetics, University Hospital Heidelberg, Heidelberg, Germany


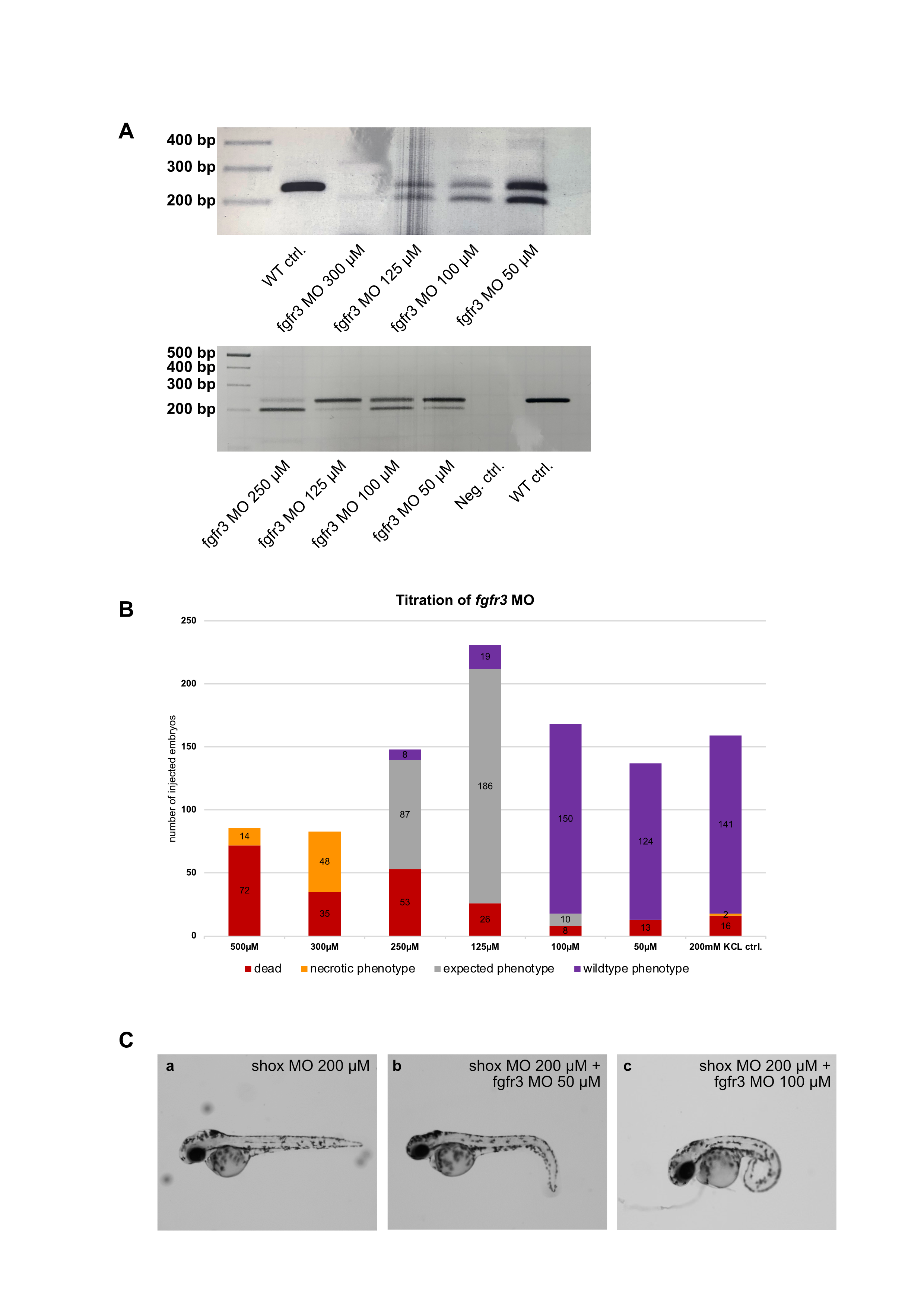


**Suppl. Fig. 1:** Validation of *fgfr3* morpholino injections. **(A)** RT-PCR analyses spanning the *fgfr3* exon-intron boundary of exon 1 to validate mis-splicing at different MO concentrations (300, 250, 125, 100 and 50 µM). The 233 bp band represents the expected wildtype product. Samples were obtained from 48 hpf embryos. **(B)** Morphological assessment of *fgfr3* MO dose titration (500, 300, 250, 125, 100 and 50 µM) to evaluate toxicity at 48 hpf (n=2-3). **(C)** *shox* single knockdown (a) and *shox*/*fgfr3* double knockdown (b,c) in zebrafish embryos 72 hpf (n=25-30 embryos). MO = morpholino; hpf = hours post fertilization; WT = wildtype.


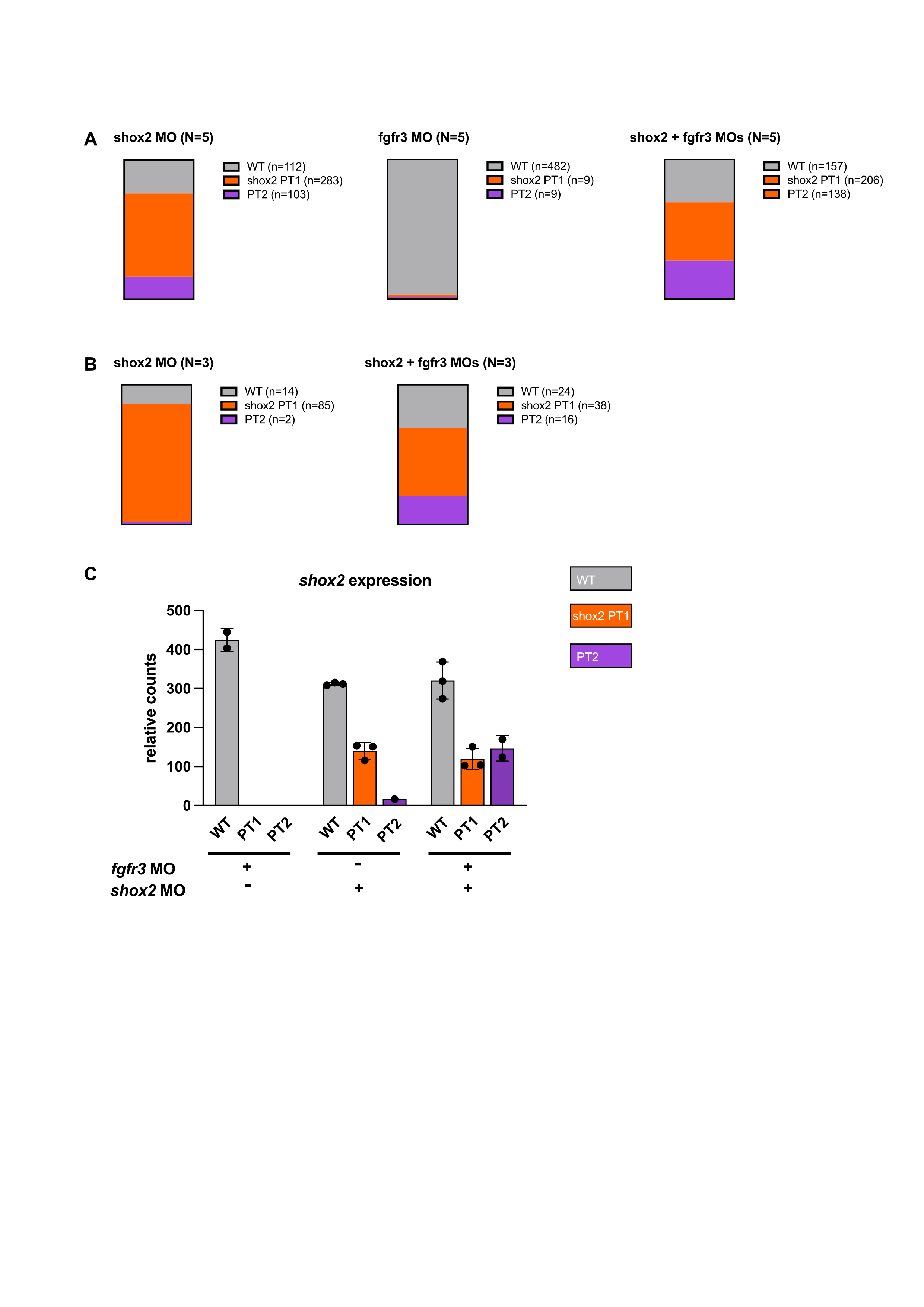


**Suppl. Fig. 2:** *shox2*/*fgfr3* double knockdown rescue experiments. Morphological assessment of **(A)** embryos injected with *shox2* (200 µM) and *fgfr3* (5 µM) single or double MOs at 72 hpf (N=5), and **(B)** embryos injected with *shox2* (200 µM) and *fgfr3* (10 µM) single or double MOs at 72 hpf (N=3). **(C)** *shox2* nCounter expression analysis in *shox2* and *fgfr3* single or double MO injected embryos at 72 hpf (N=3), 6-12 embryos were pooled per condition. MO = morpholino; hpf = hours post fertilization; N = number of independent experiments; n = number of investigated embryos; WT = wild-type; PT1 = expected shox2 phenotype (shortened fins and bradycardia); PT2 = enhanced phenotype (atrophied tail tip, severely reduced fin size, bradycardia, cerebral edema).
