# Supplementary material for: Linking shox/shox2 deficiency with fgfr3 gain-of-function and natriuretic peptides
Source: Front Endocrinol (Lausanne). 2026 Apr 17;17:1803846. doi: 10.3389/fendo.2026.1803846 (PMC13132733; doi:10.3389/fendo.2026.1803846)
Supplement: Supplementary file 2 [file Table1.docx]

| **Gene** | **Accsession number** | | **Target sequence (5' - 3')** | **Application** |
| --- | --- | --- | --- | --- |
| actb1 | NM_131031.1 | GTGCTTCTAAACAGAACTGTTGCCACCTTAAATGGCCTAGCAATGAGATTCAAACGAACGACCAACCTAAACTCTCGAACAGAACAAGATGACATCAGCA | | nCounter Control probe |
| eef1a1l1 | NM_131263.1 | GAAGGCTGCCAAGACCAAGTGAATTTCCCTCAATCACACCGTTCCAAAGGTTGCGGCGTGTTCTTCCCAACCTCTTGGAATTTCTCTAAACCTGGGCACT | | nCounter Control probe |
| rpl13a | NM_212784.1 | AAGAGAAAGGAAAAGGCCAAGCTGCGCTATTCCAAGAAGAAAGTTGAGATGAAGCTGACTAAGCAGGCTGAAAAGAACGTTGAGAGCAAGATCGCAGTAT | | nCounter Control probe |
| rps18 | NM_173234.1 | GTACAAAATCCCAGACTGGTTCCTGAACAGACAGAAGGACATAAAAGATGGGAAATACAGCCAGGTCCTTGCTAATGGTCTGGACAATAAACTGAGAGAA | | nCounter Control probe |
| b2m | NM_001159768.1 | TACTTTCGATATCAACTGCTGTTGTCCTGAATGCTGAAGGATTGTCTGCTTGGCTCTCTCGAATAAAACGGCCACAATGAGAGCACTCATCACTTTTGCA | | nCounter Control probe |
| hsp90ab1 | NM_131310.3 | CTCACAGTCCGGCGACGAGATGACCTCCCTCACAGAATACGTCAGCCGTATGAAGGAGAACCAAAAGTCCATCTATTACATCACTGGTGAGAGCAAAGAC | | nCounter Control probe |
| fgfr3 | NM_131606.2 | TTTCGAGGATGCGGGGCAATACACTTGTCTGGCAGGGAACTCGATTGGCTATAACCATCACTCTGCTTGGCTTACAGTCTTACCAGCGGTGGAGATGGAG | | nCounter target probe |
| nppa | NM_198800.3 | CTGCTGCTCCTGGTTTGGCAGCAGACGGATGTACAAGCGCACACGTTGAGCAGACACAGCTCTGACAGCAACATGGCCAAGCTCAAGAGCTTGCTGCAGC | | nCounter target probe |
| nppb | NM_001327776.1 | TAAAGTTTCTCCTTCAACGACTTGAAGAGTCCATTCCAGCTCAAGACCAAACACCGGCGGAAAGAGAAGTAAAGGCGGCAAATATTGAAGAAACCCGAGC | | nCounter target probe |
| nppc | NM_001109940.1 | CTTGTGGACTTATTCTGACTCTCCTTTCAGTCAGCACGACAGAGACTAAACCTCTGACACAGGCTGAACAGAGGTCCCTCAGGGTGCTGCTGGGAGAGGA | | nCounter target probe |
| shox | NM_001126411.1 | TTCTGGGCACCGCGAGCCACCTGGACGCGTGCAGGGTCGCTCCTTACGTGAATATGGGCGCACTCAGAATGCCATTTCAACAGGTTCAGGCTCAGTTGCA | | nCounter target probe |
| shox2 | NM_201196.1 | TCAGAACCGAAGGGCTAAATGCAGAAAGCAGGAAAATCAGTTGCATAAAGGGGTTCTCATCGGAGCGGCGAGCCAGTTTGAAGCATGTCGCGTGGCTCCA | | nCounter target probe |
| Shox MO |  | CGTGCAGAAGAAACTCACCGTCAGA | | Morpholino |
| Shox2 MO |  | AACCCCTGAAAAACAGTGATGTCTT | | Morpholino |
| Fgfr3 MO |  | AAATGAGGTGTAATGTCTGACCTGT | | Morpholino |
| Fgfr3 ex1 for |  | TTTGTGTGAGTGTGTGGCAG | | Primer mis-splice validation |
| Fgfr3 ex1 rev |  | TCCAGAAACACCTCCACCTC | | Primer mis-splice validation |

**Suppl. Table 1.** Sequences of nCounter probes, Morpholino-modified antisense oligonucleotides, and Primers
